# Early Spontaneous Abortion in Fresh- and Frozen-Embryo Transfers: An Analysis of Over 35,000 Transfer Cycles

**DOI:** 10.3389/fendo.2022.875798

**Published:** 2022-06-27

**Authors:** Jun Shuai, Qiao-li Chen, Wen-hong Chen, Wei-wei Liu, Guo-ning Huang, Hong Ye

**Affiliations:** Chongqing Reproduction and Genetics Institute, Chongqing Health Center for Women and Children, Chongqing, China

**Keywords:** early spontaneous abortion, assisted reproductive technology, fresh embryo, frozen embryo, transfer cycles

## Abstract

**Background:**

The aim of this study was to explore the risk factors for early spontaneous abortion (ESA) in fresh- and frozen-embryo transfers.

**Methods:**

This retrospective cohort study comprised a total of 35,076 patients, including 15,557 women in the fresh-embryo transfer group and 19,519 women in the frozen-embryo transfer group from January 2016 to December 2020. The primary outcome of this study was ESA, which we defined as the termination of embryonic development before 12 weeks of pregnancy (i.e., an early abortion after artificial multi-fetal pregnancy reduction was excluded).

**Results:**

In the 35,076 ART transfer cycles, the incidence of ESA was 5.77% (2023/35,076), and the incidence rates for ESA in fresh and frozen cycles were 4.93% (767 of 15,557) and 6.43% (1,256 of 19,519), respectively. Using a multivariate logistic regression analysis model, maternal age, body mass index (BMI), and number of embryos transferred were independent predictors for ESA. In addition, frozen-thawed transfer was a risk factor for ESA as compared with fresh transfer (OR = 1.207; 95% CI, 1.094–1.331; P = 0.000), blastocyst transfer was risk factor for ESA as compared with cleavage transfer (OR =1.373; 95% CI, 1.186–1.591; P = 0.000 in the total group; OR = 1.291; 95% CI, 1.111–1.499; P = 0.001 in the frozen-transfer group), and unexplained infertility was a protective factor for ESA only in the frozen group (OR = 0.746; 95% CI, 0.565–0.984; P = 0.038).

**Conclusions:**

Maternal age, BMI, number of embryos transferred, and frozen-thawed transfer were independent risk factors for ESA in assisted reproductive technology treatment cycles.

## Introduction

Assisted reproductive technology (ART) is a common option for infertility patients who wish to achieve pregnancy (1), but women undergoing ART still face many challenges in the period from clinical pregnancy to live birth—including biochemical pregnancy loss, spontaneous abortion (SA), and premature delivery. Of these, SA caused by ART has evolved into one of the greatest challenges (2). Early spontaneous abortion (ESA)—defined as a miscarriage in which the an embryo halts development prior to 12 weeks of pregnancy (3)—exhibits high incidence rates that range from 9.3% to 18.3%, and ESAs have recently been reported in ART (4, 5). Considering the economic and mental burden that infertile couples participating in ART experience with ESA, it remains uncertain whether embryo freezing improves the ESA rate compared to fresh embryos after *in vitro* fertilization (IVF). Thus, it is necessary to explore and determine the prevalence of miscarriage and its related risk factors, and provide appropriate guidance and a valuable reference for predicting the probability of ESA in ART.

In recent years there has been a sharp rise in the number of frozen-thawed embryo-transfer cycles, and although the development and maturation of embryo-freezing technology allow embryos to be frozen and stored safely for further use, it remains unclear as to whether the cryopreservation technique always provides the greatest benefit to patients (6). Several studies have revealed that compared to fresh-ET recipients, frozen-ET cycles were more likely to display a history of spontaneous abortion (SA) (7, 8); however, these studies were more concerned with live births and did not describe the factors affecting SA. In contradistinction, investigators in several other reports detected no significant difference in the risk of SA when they compared frozen-embryo transfer with fresh-embryo transfer (5, 9–12). Unfortunately, these studies did not entail subgroup analysis of SA rates according to different population characteristics, and the results may have therefore been biased. There are also some studies depicting a freeze-all strategy as associated with fewer miscarriages (13, 14).

It is not surprising to observe inconsistent results from cohort studies, which indicates that not only is a large sample size required, but that multivariate and multi-subgroup analyses are also needed. Therefore, for this study we included more than 35,000 transfer cycles by ART at our center, so as to investigate whether embryo freezing constituted a possible risk factor for ESA in a multivariate and multi-subgroup analysis approached from multiple perspectives.

## Materials and Methods

This retrospective cohort study consisted of all embryo-transfer patients at the Reproductive and Genetic Institute of Chongqing Health Center for Women and Children between January of 2016 and December of 2020. Exclusion criteria were patients with uterine malformation, chromosomal abnormality, those undergoing preimplantation genetic testing (PGT) cycles, having a history of artificial multiple-pregnancy reduction, and those who underwent oocyte-donation cycles. We ultimately included 35,076 embryo-transfer patients in our research analysis, of which 15,557 cycles were fresh-embryo transfers and 19,519 were frozen-embryo transfers. This study strictly followed the relevant requirements of the Declaration of Helsinki of the World Medical Association, and was approved by our Hospital Ethics Committee; and written informed consent was obtained from all patients at their first consultation.

The primary outcome of this study was ESA, which was defined as the stoppage of embryonic development before 12 weeks of pregnancy. Basic patient parameters included maternal age, body mass index (BMI), infertility diagnosis, ovarian-stimulation protocols (for fresh-transfer cycles), fresh/thawed-embryo transfers, endometrial preparation protocols (for frozen-transfer cycles), stage/number of embryos transferred, insemination method (for fresh-transfer cycles), and concomitant gynecological disorders.

### Statistical Analysis

Participants were first allocated to different groups according to their basic parameters, and the ESA rate was compared using the chi-squared test. We executed multivariate logistic regression to evaluate the association between the variables and ESA. All analyses were performed using IBM SPSS Statistics 21 (IBM Corp.), and all P values were two-sided, with statistical significance defined as P <0.05.

## Results

The overall incidence of ESA in the 35,076 ART transfer cycles was 5.77% (n=2023). ESA rate differed according to maternal age, BMI, infertility diagnosis (primary/secondary infertility), type of embryo transfer performed (fresh/frozen-thawed), embryonic stage (cleavage/blastocyst), and the number of embryos transferred (1, 2, or 3). In addition, in fresh-ET cycles, ESA differed among patients according to controlled ovarian stimulation (COS) protocol (GnRH agonist/GnRH antagonist, or others), and no difference was found between the IVF and ICSI insemination groups. We observed no difference in ESA rate in frozen-ET cycles among estrogen-progesterone (EP), pituitary down-regulation-EP, and natural protocols-frozen. Concomitant gynecological disorders (endometriosis/polycystic ovarian syndrome/unexplained infertility) were not different overall or in the fresh- or frozen-transfer groups (**Table 1**).

**Table 1:** Early spontaneous abortion in ART treatment cycles according to different parameters.

	Early spontaneous abortion rate						*P* (Fresh vs. Frozen)
	All	*P*	Fresh (15,557)	*P*	Frozen (19,519)	*P*	
Female age (years)		0.000**		0.000**		0.000**	
<30	4.6% (470/10289)		3.2% (157/4840)		5.7% (313/5449)		0.000**
30–34	5.2% (845/16174)		4.3% (316/7337)		6.0% (529/8837)		0.000**
≥35	8.2% (708/8613)		8.7% (294/3380)		7.9% (414/5233)		0.194
BMI (kg/m²)		0.000**		0.128		0.001**	
≤18.5	5.1% (150/2964)		3.8% (44/1166)		5.9% (106/1798)		0.011*
18.6–24.9	5.6% (1495/26618)		4.9% (585/11912)		6.2% (910/14703)		0.000**
≥25	6.9% (378/5494)		5.6% (138/2476)		8.0% (240/3018)		0.000**
Infertility diagnosis		0.000**		0.054		0.088	
Primary infertility	5.3% (924/17342)		4.7% (425/9139)		6.1% (499/8203)		0.000**
Secondary infertility	6.2% (1099/17734)		5.3% (342/6418)		6.7% (757/11316)		0.000**
Stage of embryo transfer		0.000**		0.480		0.060	
Cleavage-stage embryo transfer	5.6% (1765/31462)		4.9% (764/15516)		6.3% (1001/15944)		0.000**
Blastocyst transfer	7.1% (258/3614))		7.3% (3/41)		7.1% (255/3575)		0.964
Number of embryos transferred		0.000**		0.000**		0.045*	
One embryo	5.3% (209/3945)		4.3% (66/1521)		5.9% (143/2424)		0.033*
Two embryos	5.7% (1745/30470)		4.9% (670/13791)		6.4% (1075/16679)		0.000**
Three embryos	10.4% (69/661)		12.7% (31/245)		9.1% (38/416)		0.153
Type of transfer		0.000**	–	–	–	–	–
Fresh embryo	4.9% (767/15557)		–	–	–	–	–
Thawed embryo	6.4% (1256/19519)		–	–	–	–	–
Fresh-cycle protocols	–	–		0.004**	–	–	–
GnRH agonist	–	–	4.6% (527/11387)		–		–
GnRH antagonist/others	–	–	5.8% (240/4170)		–		–
Insemination method-fresh cycle	–	–		0.451	–	–	–
IVF	–	–	4.9% (624/12814)		–	–	–
ICSI	–	–	5.2% (143/2743)		–	–	–
Frozen-cycle protocols	–	–	–			0.704	
EP	–	–	–		6.4% (875/13588)		–
Pituitary down-regulation-EP					6.5% (368/5679)		
Natural cycle	–	–	–		5.2% (13/252)		–
Concomitant diseases, women							
Endometriosis	6.0% (311/5175)	0.380	5.0% (118/2365)	0.886	6.9% (193/2792)	0.266	0.004**
PCOS	5.7% (134/2332)	0.963	4.5% (31/689)	0.593	6.3% (103/1643)	0.775	0.094
Unexplained infertility	6.3% (86/1355)	0.351	4.2% (28/673)	0.345	8.5% (58/682)	0.025	0.001**

BMI, body mass index; GnRH, gonadotrophin-releasing hormone; IVF, in vitro fertilization; ICSI, intracytoplasmic sperm injection; PCOS, polycystic ovarian syndrome; EP, estrogen-progesterone; *P < 0.05; **P < 0.01.

In **Table 1**, the incidence of ESA in fresh and frozen cycles was 4.93% (767 of 15,557) and 6.43% (1,256 of 19,519), respectively. For the fresh-ET group, the incidence of ESA was statistically different among maternal age, protocols-fresh cycle (GnRH agonist/GnRH antagonist or others), and the number of embryos transferred (1, 2, or 3). For the frozen-ET group, ESA varied among patients according to maternal age, BMI, and the number of embryos transferred. Moreover, ESA in women aged <35 years in the frozen-transfer group was significantly elevated relative to that of the fresh-transfer group (P<0.001), and we noted no disparity with women over 35 years of age. The ESA among the three subgroups of BMI was higher in the frozen-transfer group than in the fresh-transfer group, and the ESA rate in the latter was augmented over that in the fresh-transfer group regardless of the presence of primary or secondary infertility. In the cleavage-stage embryo-transfer group, the ESA rate in the frozen-transfer group was enhanced relative to the fresh-transfer group [6.3% (1001/15944) vs. 4.9% (764/15516), P=0.000], but there was no difference with respect to the blastocyst- transfer group [7.1% (255/3575) vs. 7.3% (3/41), P=0.964]. When the number of embryos transferred was one or two, the ESA rate in the frozen-transfer group was higher than that in the fresh-transfer group, but there was no difference when three embryos were transferred. Among the three subgroups of women with concomitant disease such as endometriosis or unexplained infertility, the ESA rate was higher in the former than in the latter group. Nevertheless, we observed no difference the ESA rate in young women (<35 years) between the diminished ovarian reserve (DOR) group and non-DOR group (P>0.05), and advanced age women (≥35 years), consistent results were also observed. After stratification according to fresh embryo and frozen embryo, there was still no statistical difference in ESA rate between the DOR group and the non-DOR group (P>0.05). However, after stratification according to the DOR and non-DOR, the ESA rate of the advanced age group was significantly higher than that of the younger group (P<0.05) (**Table S1**).

In **Table 2**, all of the ESA-related factors depicted above were re-analyzed simultaneously using a multivariate logistic regression analysis model with adjusted data. In the total and fresh- and frozen-transfer groups, maternal age, BMI, and the number of embryos transferred were independent predictors of ESA. In addition, frozen-thawed transfer was a risk factor for ESA as compared with fresh transfer (OR = 1.207; 95% CI, 1.094–1.331; P = 0.000). In both the total and frozen-transfer groups, blastocyst transfer was a risk factor for ESA compared with the transfer of cleavage-stage embryos (OR =1.373; 95% CI, 1.186–1.591; P = 0.000 in the total group and OR =1.291; 95% CI, 1.111–1.499; P = 0.001 in the frozen-transfer group); while unexplained infertility was a protective factor with regard to ESA only in the frozen-transfer group (OR = 0.746; 95% CI, 0.565–0.984; P = 0.038).

**Table 2:** Factors associated with ESA rate using logistic regression analysis.

	All (35,076)		Fresh (15,557)		Frozen (19,519)	
	Adjusted OR (95% CI)	*P*	Adjusted OR (95% CI)	*P*	Adjusted OR (95% CI)	*P*
Female age	1.044 (1.035–1.054)	0.000**	1.084 (1.067–1.102)	0.000**	1.026 (1.014–1.037)	0.000**
BMI (kg/m^2^)	1.035 (1.019–1.052)	0.000**	1.028 (1.002–1.056)	0.035*	1.041 (1.020–1.062)	0.000**
Type of transfer (frozen/fresh)	1.207 (1.094–1.331)	0.000**	-	-	-	-
Stage of embryo (blastocyst/cleavage)	1.373 (1.186–1.591)	0.000**	-	-	1.291 (1.111–1.499)	0.001**
Number of embryos transferred	1.295 (1.141–1.469)	0.000**	1.326 (1.074–1.637)	0.009**	1.239 (1.058–1.450)	0.008**
Unexplained infertility	-	-	-	-	0.746 (0.565–0.984)	0.038*

BMI, body mass index; *P < 0.05; **P < 0.01.

## Discussion

The use of fresh-embryo transfers in relation to frozen transfers is diminishing readily worldwide. Freeze-all embryo protocols followed by elective frozen-embryo transfer constitute an effective method for the prevention of ovarian hyperstimulation syndrome (OHSS), as a recent Cochrane review by Zaat and co-authors showed that the risk of OHSS was reduced by 75% with their freeze-thawing procedures (15). Frozen-embryo transfer also significantly lowers the risk of a baby being born small-for-gestational age (SGA) or with a low birthweight compared to babies born from fresh transfers, as shown in the systematic review by Maheshwari and co-workers in 2018 (16). Several randomized controlled trials (RCTs) have shown that frozen- embryo transfer portends better pregnancy and live-birth rates than with fresh-embryo transfer (17–20). Frozen-embryo transfer is also being increasingly used in clinical practice instead of fresh embryo transfer due to the benefits reported above, but whether this relatively new transfer strategy affects ESA is presently unknown; and there is a current lack of studies that entail both a large sample size and multi-dimensional research.

Infertile women today face tremendous biological and societal pressures, and a miscarriage during ART treatment further aggravates their psychological and financial stresses (21). It is conventional scientific knowledge that advanced age and obesity in women increase their risk of ESA, and our research reached the same conclusion; but whether frozen-embryo transfer will ultimately increase ESA rates remains controversial, and whether it can bring added benefit has aroused the concerns of both physicians and patients (22, 23). Our study first showed that the incidence of ESA in overall ART transfer cycles was 5.77%, that in fresh- and frozen-embryo transfer cycles the rates were 4.93% and 6.43%, respectively; and that the frozen-embryo transfer rate was significantly higher than in fresh-embryo transfers. Investigators uncovered an early miscarriage rate as slightly higher in women conceiving with frozen-embryo transfer compared with those conceiving with intrauterine insemination (IUI) (24). IUI, which only involves sperm preparation and/or ovulation induction, emulates natural conception more closely. The disparity in ESA rates between frozen-embryo transfer and IUI cycles highlights the impact of embryonic *in- vitro* manipulation on ESA, and the distinction in ESA between frozen-embryo and fresh-embryo transfer cycles also further accentuates the effects of the *in vitro* manipulation of frozen-thawed embryo on ESA. However, we need to further consider that the different population characteristics may have interfered with our results.

In view of the basic patient characteristics, we explored the impacts of maternal age, BMI, and infertility diagnosis on ESA with respect to the total ART transfer cycles, as well as on the fresh-embryo and frozen-embryo transfer subgroups. Our data revealed that women of advanced maternal age, who were obese, and/or with secondary infertility were at greatest risk of ESA in the 35,076 ART transfer cycles; and this was consistent with previous findings (25–27). Whereas only advanced maternal age was correlated with a greater risk for ESA in the fresh-embryo and frozen-embryo transfer subgroups, only obese women in the frozen-embryo transfer subgroup exhibited a greater risk of ESA. This suggests that age, obesity, and frozen-thawed embryo protocols adversely impact ESA rates collectively. Nevertheless, we observed no difference the ESA rate in young women (≤35 years) between the DOR group and non-DOR group, and advanced age women (>35 years), consistent results were also observed. After stratification according to fresh embryo and frozen embryo, there was still no statistical difference in ESA rate between the DOR group and the non-DOR group. However, after stratification according to the DOR and non-DOR, the ESA rate of the advanced age group was significantly higher than that of the younger group. These results suggest that women of advanced age rather than DOR increase ESA rates. In the fresh-embryo transfer subgroup, the ESA rate in GnRH antagonist (GnRH-ant) or other COS protocols was significantly higher than in GnRH agonist (GnRH-a) protocols, which was also congruent with previous RCT findings; but this may have also been due to the small sample size in the aforementioned study (28). However, a previous multivariate logistic regression analysis of 18,853 patients recruited to our Center revealed that the cumulative live-birth rate in the GnRH-ant group was lower than that in the GnRH-a group (OR=2.11; 95% CI, 1.69–2.63), particularly with respect to the suboptimal ovarian responders (where 4–9 oocytes were retrieved) (29). In another retrospective analysis, authors compared the efficiency of the GnRH-ant protocol with that of the GnRH-a protocol in patients with DOR, and demonstrated that the former possessed a lower ET-cancellation rate and higher implantation rate than the latter (30). Our data revealed that a GnRH-a protocol may be superior to a GnRH-ant protocol in terms of ESA rates, and from their different outcome indicators the previous authors suggested that GnRH-a protocols may relate to improved embryo quality or endometrial receptivity; this, however, warrants further investigation. The ESA rate for blastocyst transfers was markedly higher than for cleavage-stage embryo transfers, but we noted no difference in the subgroup analysis of fresh and frozen cycles—which was inconsistent with previous studies (31, 32). This discrepancy may be caused by data bias between the groups, and the data divergence between the groups therefore needs to be further adjusted to the numerical disparities; and we need to balance the confounders between groups. Needless to say, the higher the number of embryos transferred, the higher the abortion rate. And ESA rates with respect to the insemination method were indistinguishable between fresh-embryo transfer and frozen-embryo transfer cycles (i.e., estrogen-progesterone/natural cycles), which agreed with previous findings (33). Recognizing that some concomitant diseases in women who undergo ART may also affect ESA, we selected endometriosis, PCOS, and unexplained infertility as representatives for our subgroup analysis; and uncovered no statistical difference in ESA rates among the total, fresh-, and frozen-transfer groups. However, ESA was higher in the frozen-transfer group than in the fresh-transfer group regardless of the presence of endometriosis or unexplained infertility. This suggests that embryo freezing itself increases the ESA rate after excluding the influences of the aforementioned accompanying diseases.

Another intriguing principal finding from the current study was that the ESA rate in women under 35 years of age in the frozen-transfer group was significantly higher than that in the fresh-transfer group, while the difference was not statistically significant for women over 35. This indicated that by excluding the influence of advanced reproductive age on abortion, embryonic freezing itself still increased ESA rates. In the BMI subgroup analysis, we also ascertained that by discounting the BMI subgroup, the ESA rate of the frozen-embryo group was still higher than that of the fresh embryo-transfer group, indicating that after excluding the influence of BMI on abortion, embryo-freezing technology still increased the risk of miscarriage (7, 8). The same results were also shown in the subgroup analysis of infertility diagnosis and the number of transferred embryos. In addition, multivariate logistic regression analysis further confirmed the above conclusion: that compared with fresh-embryo transfer, frozen-thawed-embryo transfer was an independent risk factor for ESA. The novelty of the present study, then, was our analysis from the perspective of multiple dimensions that showed that the ESA rate with frozen-embryo transfer was higher than that with fresh-embryo transfer, while ESA was observed to be either higher or not different between fresh-embryo transfers and frozen-embryo transfers in the majority of the previous studies (9–14). We posit that several reasons may explain the discrepancy among studies. First, most investigators—including ourselves—did not exclude all possible confounding factors related to ESA in their analyses. In addition, small-sample, single-center cohort studies may generate data bias, while large-sample cohort studies contain data from multiple centers or are performed over long periods of time where the methods of embryo cryopreservation, embryo thawing, and *in vitro* culture may have changed dramatically during follow-ups. These bias effects are difficult to avoid in actual work, so it is challenging to strictly control confounding factors in real-world investigations. Finally, it is unfortunate that our study lacked data on whether patients experienced a spontaneous abortion before undergoing ART; this partially limited our conclusions, as patients with a history of spontaneous abortion may manifest a higher risk of recurrent spontaneous abortion.

## Conclusions

This cohort study confirmed that the transfer of frozen embryos increased the risk of ESA during ART cycles relative to fresh embryos. In addition, maternal age, BMI, and number of embryos transferred also increased ESA. Therefore, frozen-transplantation strategies need to be more cautiously assessed so as to provide patients with the greatest benefits possible with respect to safety.

## Data Availability Statement

The raw data supporting the conclusions of this article will be made available by the authors, without undue reservation.

## Ethics Statement

The studies involving human participants were reviewed and approved by Institutional Review Board (IRB) of Chongqing Health Center for Women and Children. The patients/participants provided their written informed consent to participate in this study.

## Author Contributions

JS designed the cohort; JS, Q-LC, W-HC, W-WL, G-NH, and HY conducted the trial; JS and Q-LC executed the statistical analyses and prepared the tables with oversight by HY; and JS, Q-LC, and WHC drafted the manuscript. All authors were involved in the data collection, interpreted the data, provided critical input to the manuscript, and approved the final submitted version.

## Funding

This study was supported by a grant from the Medical Research Foundation of Chongqing to Qiao-Li Chen (grant number 2020MSXM090).

## Conflict of Interest

The authors declare that the research was conducted in the absence of any commercial or financial relationships that could be construed as a potential conflict of interest.

## Publisher’s Note

All claims expressed in this article are solely those of the authors and do not necessarily represent those of their affiliated organizations, or those of the publisher, the editors and the reviewers. Any product that may be evaluated in this article, or claim that may be made by its manufacturer, is not guaranteed or endorsed by the publisher.
