# Supplementary material for: Early Spontaneous Abortion in Fresh- and Frozen-Embryo Transfers: An Analysis of Over 35,000 Transfer Cycles
Source: Front Endocrinol (Lausanne). 2022 Jun 27;13:875798. doi: 10.3389/fendo.2022.875798 (PMC9271787; doi:10.3389/fendo.2022.875798)
Supplement: Supplementary file 1 [file Table_1.docx]

**Supplementary Table 1. Early spontaneous abortion in ART treatment cycles according to female age and DOR**

|  | **Early spontaneous abortion rate** | | | | |
| --- | --- | --- | --- | --- | --- |
|  | **Female age <35 years** | ***P*** | **Female age ≥35 years** | ***P*** | ***P*^a^** |
| All |  | 0.207 |  | 0.509 |  |
| DOR | 4.3%(77/1774) |  | 8.7%(100/1147) |  | 0.000** |
| Non-DOR | 5.0%(1238/24688) |  | 8.1%(608/7466) |  | 0.000** |
| Fresh |  | 0.666 |  | 0.468 |  |
| DOR | 3.6%(35/965) |  | 9.6%(41/426) |  | 0.000** |
| Non-DOR | 3.9%(438/11211) |  | 8.6%(253/2954) |  | 0.000** |
| Frozen |  | 0.383 |  | 0.771 |  |
| DOR | 5.2%(42/809) |  | 8.2%(59/721) |  | 0.019* |
| Non-DOR | 5.9%(800/13477) |  | 7.9%(355/4512) |  | 0.000** |

Note: DOR, diminished ovarian reserve; *P*^a^, Female age **<**35 years vs. Female age ≥35 years; *, *P* <0.05; **, *P* <0.01.

**Supplementary Table 2. Early spontaneous abortion in female age ≥35 years**

|  | **Early spontaneous abortion rate** | | | | | | ***P* (Fresh vs. Frozen)** |
| --- | --- | --- | --- | --- | --- | --- | --- |
|  | **All** | ***P*** | **Fresh**(15,557) | ***P*** | **Frozen**(19,519) | ***P*** |  |
| Female age (years) |  | 0.535 |  | 0.225 |  | 0.826 |  |
| 35-39 | 8.1% (435/5385) |  | 8.4%(210/2514) |  | 7.8%(225/2871) |  | 0.488 |
| ≥40 | 8.5%(273/3228) |  | 9.7%(84/866) |  | 8.0%(189/2362) |  | 0.124 |
